# Natural Product Medicines for Honey Bees: Perspective and Protocols

**DOI:** 10.3390/insects10100356

**Published:** 2019-10-18

**Authors:** James P. Tauber, William R. Collins, Ryan S. Schwarz, Yanping Chen, Kyle Grubbs, Qiang Huang, Dawn Lopez, Raymond Peterson, Jay D. Evans

**Affiliations:** 1 USDA-ARS Bee Research Laboratory, BLDG. 306, RM. 315, BARC-EAST, Beltsville, MD 20705, USA; judy.chen@ars.usda.gov (Y.C.); kyle.grubbs@ars.usda.gov (K.G.); Dawn.Lopez@ars.usda.gov (D.L.); raymond.peterson@granitepv.com (R.P.); 2 Department of Chemistry, Fort Lewis College, 1000 Rim Drive, Durango, CO 81301, USA; collins_w@fortlewis.edu; 3 Department of Biological Sciences, Fort Lewis College, 1000 Rim Drive, Durango, CO 81301, USA; rsschwarz@fortlewis.edu; 4 Honeybee Research Institute, Jiangxi Agricultural University, Zhimin Avenue 1101, Nanchang 330045, China; qiang-huang@live.com; 5 Granite Point Ventures LLC, 10 Lakeview Circle, Greenbelt, MD 20770, USA

**Keywords:** honey bees, bee disease, colony loss, natural product, traditional medicine, screening, virus

## Abstract

The western honey bee remains the most important pollinator for agricultural crops. Disease and stressors threaten honey bee populations and productivity during winter- and summertime, creating costs for beekeepers and negative impacts on agriculture. To combat diseases and improve overall bee health, researchers are constantly developing honey bee medicines using the tools of microbiology, molecular biology and chemistry. Below, we present a manifesto alongside standardized protocols that outline the development and a systematic approach to test natural products as ‘bee medicines’. These will be accomplished in both artificial rearing conditions and in colonies situated in the field. Output will be scored by gene expression data of host immunity, bee survivorship, reduction in pathogen titers, and more subjective merits of the compound in question. Natural products, some of which are already encountered by bees in the form of plant resins and nectar compounds, provide promising low-cost candidates for safe prophylaxis or treatment of bee diseases.

## 1. Introduction

The European honey bee (*Apis mellifera*) is the single most important managed pollinator of fruit and vegetable crops worldwide. In the United States, honey bee pollination supports crops with a worth approaching $15 billion annually. These include nuts like almonds, macadamia and other high-value nut crops; tree fruits like apples, cherries, oranges, peaches and grapefruit; berries like strawberries and blueberries; and row crops such as cucumbers, melons, pumpkins and cantaloupe. All these crops, and more, depend 80% to 100% on the honey bee for pollination. Diverse bee species, other insects, birds and bats also contribute to crop pollination (https://www.uaex.edu/farm-ranch/special-programs/beekeeping/pollinators.aspx). Loss of pollination services from honey bees could be unsustainable because, in this scenario, countries would need large expansions of agricultural cultivated land, thus further destroying surrounding ecosystems and likely with increased costs to farmers [1].

Colony loss is a severe threat to honey bee populations and the pollination services they provide, along with the production of honey, wax and other hive products. Current average annual colony losses for local U.S. beekeepers with fewer than 50 reported hives in the U.S. is 50.9% (https://bip2.beeinformed.org/survey/). Commercial beekeepers (500+ hives) also reported significant annual losses (32.9%). Loss of this magnitude far exceeds the 20th century historic rate of 5% to 10% and costs the beekeeping industry $250M annually in replacement and lost productivity. Despite overwintering losses, there has generally been a worldwide increase in the number of managed colonies, and beekeepers can compensate for losses by splitting surviving colonies or by purchasing honey bees [1]. Whether or not this is a long-term sustainable practice remains a concern.

Factors that may negatively impact honey bee health include queen failure; pesticides; lack of suitable foraging land; drought; overwintering losses; pathogens that are transmitted between colonies, within colonies, via flowers and/or pests; and/or possibly opportunistic microorganisms. Of these, diseases are the most frequently cited factor in honey bee decline. Fortunately, alongside replacing queens more frequently, providing diverse edge crops in agricultural fields, and/or reducing pesticide exposure, there are many avenues for reducing the impacts of disease. Our premise is that against any background, colonies are weaker due to diseases, which increases individual mortality and reduces foraging, thus the colonies are at a higher risk for extinction [2]. Therefore, we examine key pathogens that are negatively associated with honey bee health, current ideas on treating or preventing these diseases and put forth ideas and protocols to discover and test honey bee medicines with the overarching goal to support honey bee populations.

### 1.1. Key Agents of Disease

In both Europe and the U.S., honey bee declines and colony loss are closely tied to the mite *Varroa destructor* [3]. This pest feeds on both pupal and adult honey bees. Mite parasitism weakens bees and shortens lifespans, causing lower individual contribution to the hive. One consequence of shorter lifespan is reduced colony productivity when reproduction is unable to keep pace with loss and colony census declines. The *Varroa* mite is also pernicious because of the viruses it carries and transmits to honey bees, including the RNA virus, deformed wing virus (DWV), and its variants [4,5]. The honey bee is afflicted by other viruses as well; scientists have identified nearly 20 virus species in U.S. honey bees alone and the list is growing. Researchers continue to screen honey bee colonies for emerging or novel viruses using advanced DNA and RNA sequencing technologies [6]. Infection with any of these viruses beyond a certain level is likely to have a negative effect on bees, hive productivity and colony fate, leading to additional costs for beekeepers and pollination services. Maladies of the gut represent another frequent class of honey bee disease. Nosemosis, caused by the gut parasites *Nosema ceranae* or *Nosema apis*, is found worldwide. Similarly, there are more recently highlighted gut diseases like that caused by the trypanosomatid gut parasites, e.g., *Lotmaria passim*. All have been tied to poor colony growth and higher mortality [7]. A further concern for bee health is dysbiosis, a disruption of the natural gut microbiota [8,9]. Bacterial symbionts in the honey bee intestine are important for nutrient homeostasis and protection against pathogens, and disruption of the microbiota can make bees more susceptible to opportunistic pathogens, including trypanosomatids and the bacterium *Serratia* [10,11].

### 1.2. Disease Costs for Beekeeping

The ever-increasing cost paid by beekeepers to create and maintain healthy colonies is driving the increase in pollination fees paid by almond growers and others. Almond pollination fees in the U.S. have tripled since 2004, from $60/hive in 2004 to $192/hive in 2019, largely in response to the expense of colony loss (https://www.thebeecorp.com/thebeeword/2019-almond-pollination-prices). Immediate disease expenses for beekeepers run into the hundreds of millions of dollars annually when products, management costs and lost pollination opportunities are included. As one example of management cost for a commercial beekeeper, a one-time registered fall treatment against *Varroa* mite for 120 remaining colonies before overwintering costed $1397 ($1248 for materials, $121 for labor and $28 for transportation), which comes to about $11.64 per colony [12]. Many beekeepers apply several treatments per year against several diseases and pests, thus making for a potential cost of $50 or more per hive. Additionally, certain products cannot be administered at any time out of concern that the compound will be stored in commercialized honey or wax and thus possibly unsafe for human consumption.

Because of all the factors outlined here, there is a critical need for safe medicines that a beekeeper can easily, safely and possibly at any time of the year apply to the colony to improve honey bee resilience to pathogens and increase bee lifespans. Having a registered treatment is especially important because this indicates that scientific experimentation has verified the product’s stated safety and effectiveness claims. The need for new or improved honey bee treatments is unlikely to go away because of the never-ending evolutionary arms race between pests and the means used by bees and beekeepers to control them. This is especially true in the U.S., where genetic variability of honey bee populations is relative reduced compared to that in Europe [1]. Furthermore, in addition to resistance by known threats, honey bees are subject to host jumping, introduction of foreign pathogens, or emergence of novel pathogens that can overcome current efforts.

### 1.3. Registered Bee Disease Treatments

Natural products are chemicals that are found in nature and produced from living organisms, often plants or microbes. In contrast, a synthetic treatment arises when a chemist invents an entirely new synthetic structure or, more often, when the chemist creates an analogue of a natural compound. More commonly, in the pharmaceutical industry, tests are done by screening existing panels of small molecules and chemicals that are already known and exist (e.g., the ‘Generally Recognized as Safe’ (GRAS) panel). Either way, a synthetic compound is one that is not produced in nature, but may be modeled from nature, and chemical testing generally starts with a library of already known compounds.

As natural product examples, one natural product that has received registration to combat *Varroa* is oxalic acid. Oxalic acid (OA) derives from the wood-sorrel (*Oxalis*) flowering plant. In the U.S., OA is the most recently registered honey bee product against mite parasites. Another natural product used in *Varroa* control is thymol, marketed under a variety of trade names, including Apiguard. Additionally, HopGuard^®^ is a mix of beta-acids derived from the beer brewing process and hop plants, and is also labeled and marketed for mite control.

In contrast, current registered synthetic products for honey bee health in the U.S. include a variety of acaricides aimed at parasitic mites (e.g., organophosphates, pyrethroids and formamidine pesticides [13]) and antibiotics used in the U.S. for the control of American and European Foulbrood, which are two highly infectious bacterial disease agents [14]. Fumagillin is also a synthetic product registered for *Nosema* control [15]. It is, however, a naturally produced toxin derived from the fungus *Aspergillus fumigatus* [16]. Although it is a labeled product, its continued use is in question due to a lapse in production. Moreover, recent indications are that it may not be highly effective against *Nosema ceranae*, a species which has largely replaced *Nosema apis* [15]. We discuss below the current state of the art for treatments against *Nosema*.

### 1.4. Why Natural Products?

Numerous studies in the medical literature suggest that natural products can reduce viruses and bacteria [17,18,19,20]. Natural products are the basis of traditional Chinese medicine. Likewise, the European pharmacopeia was exclusively plant-based throughout and after the Renaissance. Natural products constitute the active ingredient in many medicines found in households. Examples include aspirin (salicylic acid) for pain relief and inflammation; opiates for pain relief; quinine (Qualaquin^®^) to combat malaria; penicillin as an antibiotic; digoxin for heart failure and cardiac arrhythmias; and paclitaxel (taxol) for cancer.

Many beekeepers appear to favor a natural approach to medicines, as this methodology is aligned with the naturalistic ideals favored by many in this community. Beekeepers generally have no desire to contribute to antibiotic resistance, to pollute the environment nor to feed synthetic chemicals to their hardworking bees. Although both synthetic and natural products can be equally “harsh,” the idea to apply naturally made compounds from naturally assembled substances (e.g., propolis that contains plant-derived natural products) is an attractive putative medicine. Here, we define natural products as compounds produced from living organisms, and we include the subset of these compounds that can be produced in larger quantities by other means (which produce the exact same compound). Most importantly, chemicals used to treat bees may sometimes find their way into wax or honey, tainting “all-natural products” that people use or consume. Thus, people may contact or consume any chemical that the beekeeper uses to treat a colony. Many natural products are safe when consumed in reasonable quantities. The U.S. Food and Drug Administration (FDA) has regulatory oversight over food and medicines and has a special category named ‘Generally Recognized as Safe’ (GRAS) for natural products that people regularly consume in small amounts (https://www.fda.gov/food/food-ingredients-packaging/generally-recognized-safe-gras). A GRAS compound has a simple and inexpensive regulatory pathway when the dose is no more than what the FDA recognizes as safe. This contrasts with non-GRAS natural and synthetic compounds which may require human clinical trials to demonstrate safety for people. The expense of such trials often exceeds the perceived market for honey bee medicines.

Empirically or anecdotally, many GRAS compounds are recorded to be effective against viruses, bacteria, and eukaryotic parasites in mammals and insects, or may improve (perceived) overall health. The strategy to select GRAS products and compounds that are implicated in enhanced immunity or pathogen reduction in humans, other mammals, or other insects is sound from evolutionary first principles.

### 1.5. Current Research on Natural Products for Bee Disease

Scientists and beekeepers alike are keen to find new natural products that improve bee health. However, there is an initial hurdle to narrow the universe of tens of thousands of natural products to a number that is tractable to study. One way to get over this hurdle is to “combine” naturally produced compounds by using an assemblage of them, e.g., propolis that is made by bees and is composed of substances from both plants and bees, or crude extracts from plants or mushrooms. Individual compound testing and testing crude extracts have already shown several promising avenues. Marla Spivak and collaborators have demonstrated significant disease reduction by naturally collected or added propolis (plant resins) in the hive environment [21,22,23]. Nectar and pollen phytochemicals under controlled experiments also provided evidence that these naturally occurring honey bee foods support honey bee health [24]. Silvio Erler and colleagues have shown promising effects of extracts from Bay laurel on honey bee pathogens [25], and suggested that bees might self-medicate with specific plant products [26,27]. In this work, the self-medication on a colony-level appeared to be behavioral and social (*i*.*e*., superorganismal), and pathogen-specific. Polyphenols [28,29] and alkaloids [30] could help fight infections in honey bees. Finally, extracts from polypore mushrooms appeared to be effective against RNA viruses. In a recent study, honey bees infected with two positive-strand RNA viruses, deformed wing virus and Lake Sinai virus, were fed either polypore mushroom extracts dissolved in sugar water or sugar water without the mushroom extract as the control. The bees that were fed the mushroom extracts showed reduced virus levels [31]. Andre Burnham provides an excellent, recent summary of the various treatments against the gut parasite *Nosema*, which includes natural products (individual and from crude extracts), RNAi methods and probiotics [32]. As one example of a natural product, modified porphyrins, which are produced by living organisms and also synthetically, deform the cell wall of *Nosema*, significantly reduce spore loads in bees, and increase survival of infected bees yet do not increase mortality of non-infected bees [33]. The experimental design was a rational approach, namely that the researchers considered novel natural products to combat a honey bee disease, identified a compound based on its efficacy against pathogens in other models and then carried out experimentation in bees. A team at the University of Alberta is now experimenting to produce large quantities of this compound in a lab synthesis project, aiming for colony-level treatments against *Nosema* (http://2018.igem.org/Team:UAlberta/Description).

### 1.6. RNA Interference

One might argue that RNA interference (RNAi) is a natural product because it is a natural immune defense against virus infection across multiple kingdoms of life. The RNAi mechanism acts to suppress viral infection by disrupting translation of viral messenger RNA transcripts. For several years, scientists were optimistic that RNAi would make for safe and effective control of honey bee viruses [34,35,36]. While this optimism has not resulted in marketable products, work continues to improve upon prior results. One challenge with RNAi is the ability of highly variable RNA viruses to evade and even counterattack the honey bee RNAi response. Recent work showed that colony infection by DWV involves a variable population of DWV [37]. These viruses differ in sequence due to mutation and recombination. Diversity in DWV transcripts likely reduces the chance that candidate RNAi guide strands have exact matches to every virus transcript in the population. Imperfect hybridization reduces RNAi efficiency, and thus the efficiency of RNAi as a treatment. A further impediment is that scientists have found it difficult to deliver a sufficient quantity of guide RNAi strands to cells that harbor the virus [35], especially for colony-level implementation.

### 1.7. High-Throughput Screening in the Laboratory

One way to potentially screen many compounds quickly is to culture pathogens in the laboratory without bee hosts. Microbes such as *Serratia* spp. and the causative agents of honey bee foulbrood and chalkbrood are readily cultured in the laboratory and, as such, these are targets for high-throughput screening of natural products. Recent work has demonstrated methodologies and some promising candidates targeting these disease agents [38,39,40]. This research and methodology complement efforts to identify biologics, such as bacteria [41,42] or bacteriophages [43,44], that impact key honey bee pathogens in culture.

With the availability of a honey bee embryonic cell line [45], it is also possible to use in vitro screens of candidate treatments for key bee diseases. Indeed, this cell line has been shown to harbor and support honey bee viruses [46], opening the door to direct antiviral screening in a cell-based system. Similarly, the microsporidian *Nosema ceranae*, an obligate parasite of honey bee gut cells, is currently screened mainly in live honey bees, but studies can also be performed in non-bee cells lines which are more readily available to non-bee labs [47]. However, cell lines have severe limitations and require extremely sterile conditions. If this parasite can also be established in stable honey bee cell lines, future screening for treatments against this key disease will be vastly simplified, but there is a disconnect between the minute observances seen from cell line studies compared to those from live animals. Someday there may be a balance between animal-pathogen research that combines cell lines and live animal trials in order to reduce the number of live animals needed for an experiment.

Many, but not all, human drugs are designed to interact with a specific target protein. If a specific protein from honey bee pathogens were identified, a high-throughput protein expression assay to screen compounds could be developed. To our knowledge, there is no protein expression assay for proteins from a honey bee pathogen. Likewise, we do not know of a protein expression assay for honey bee immunity proteins, which could be used to identify candidates for changing their expression levels.

For drug discovery, it is also possible to perform virtual computational screens. In this method, one needs a three-dimensional structure of the target, a three-dimensional library of chemical compounds, modeling software to screen the library to the target and computing power [20]. Screening libraries consisting of thousands, and up to hundreds of thousands, of compounds are readily available, as is the needed computational support. Intriguingly, there are three-dimensional structures available for the capsid (protein containers) for honey bee viruses [48,49]. Scientists, in general, have been successful in designing small-molecule drugs that interfere with virus capsids. Thus, a computational screen of the bee viruses may identify one or more chemical structures that weakly or strongly bind to these capsids. These chemicals could become honey bee medicines or may become medicines after acquiring sufficient drug properties through chemical adjustment(s).

### 1.8. Limits of Approach

A key question in studies to combat diseases is ‘do reduced pathogen and parasite loads lead to improved honey bee health?’ We anticipate that natural products that significantly reduce viral or parasite loads will causally improve hive health. However, this is not a given. A compound that significantly decreases viral or gut parasite load may have a positive, negative, or no effect on hive health. Indeed, improving hive rather than solely individual bee health is the ultimate goal. It is also likely that improving honey bee health is more reliant on better apiary management and practices which include education and awareness campaigns, improving land management practices, or monitoring climate effects on pollinator survivability. Finally, it may prove that the natural product approach may find a natural antibiotic or immune stimulant, but one that has only weak effect(s). To achieve enough potency, it may be necessary to perform medicinal chemistry on the identified compound to synthetically adjust its structure for gained potency. In this situation, the compound is no longer a natural product but rather a synthetic analogue that may require additional screening and tests to be considered safe. Alternatively, inspiration from synthetic adjustments may lead to finding the same compound produced from a living organism.

### 1.9. Goal of This Review and Protocol Description

**Figure 1:**
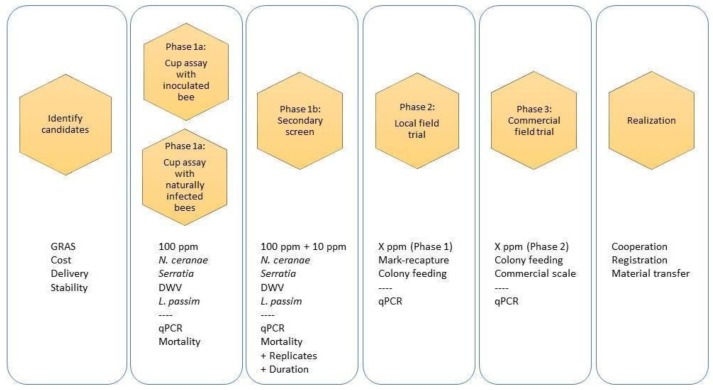
Natural product screening process.

This paper will describe infrastructure for the screening and development of natural bee medicines in *living* bees. The Bee Research Laboratory of the USDA Agricultural Research Service and others have developed efficient and cost-effective live-bee screening techniques. However, there is currently not a standard pipeline to (1) identify natural product candidates for testing; (2) perform high-throughput genetic or physical screens; and then, (3) conduct practical field trials for simultaneous testing of multiple new medicines for bees. Here, we propose such a pipeline. Detailed protocols and shared metrics are the first steps towards harmonization of community activities. In this way, the protocols we described and referenced here may contribute to or motivate the harmonization of honey bee research protocols as done in the COLOSS BEEBOOK series (https://coloss.org/core-projects/beebook/). Our objective is to promote the sharing of methods, measures, data format, analysis and metrics so that hard work among the community leads to reports that are congruent, built on one another, and advance the shared interest of preventing and curing disease in honey bees. Figure 1 presents a diagram of our process to identify, prioritize, screen, field test and register natural medicines. We recognize and note that written protocols have many limitations. It is often time consuming to prepare them, the protocols themselves often struggle for completeness or lag advances, they are not universally effective for all skill and experience levels, and they require iterative refinement based on feedback from many new users of varying skill levels. We suggest that the following written procedures are a testament to the demand for new, natural cures to combat lingering (and novel) honey bee health issues, and these protocols will benefit from iterative refinement and companion instructional videos.

## 2. Protocols

### 2.1. Selection Criteria

The first task of drug discovery is to compile a list of natural products that are generally recognized as safe (e.g., GRAS) in the U.S. or another country, and are potential immune stimulants, antiviral, or antiparasitic in animals, honey bees or simply in vitro. Potential starting points to create this candidate list include:

- Plant nectar products [24];
- Plant resins (and compounds derived from plant resins), especially those collected by bees in the field [23];
- Natural products proposed by community members and tested under material transfer and research agreements (e.g., polypore mushroom extracts developed by Fungi Perfecti [31] and commercialized probiotics);
- FDA GRAS database and lists of natural extracts vetted against peer-reviewed literature (known antiviral and antiparasitic activity);
- Compounds known from the insect literature to be antiviral/antimicrobial/antiparasitic;
- Compounds known from general literature to be antiviral/antimicrobial/antiparasitic;
- Herbs with potential health benefits in other organisms [25];
- The NCI Natural Products Repository (https://dtp.cancer.gov/organization/npb/introduction.htm) that includes extracts from 80,000 plants that are native to Africa and Madagascar, Central and South America, and Southeast Asia; 20,000 specimens from marine invertebrates and algae from the southern oceans; and specimens from 16,000 microbes.

Additional criteria or down-selection filters include:

- Projected maximum product cost of $5 USD per treatment (approximately half the cost of one current treatment);
  ○ The natural product in question may end up being more expensive, and then the choice of which treatment to apply will be based on other criteria;
  ○ Reliability of production (e.g., synthetic compounds inspired from naturally produced compounds);
- Chemoinformatic properties that suggest favorable target interaction:
  ○ Chemical diversity across terpenoids, carbohydrates, sterols, alkaloids, lipids, small-molecule secondary metabolites and extracts, especially when compounds lack a report of a molecular target [17];
  ○ Stability at circa 35 °C with high relative humidity (50%–90%), and solubility in high-osmolarity sugar-water solution for administration;
- Consideration of cross-contamination into honey bee products and to flowers and other commercial and wild insects;
- Compounds that are reported to provide therapeutic effect at a dose of 1 ppm, 10 ppm, or 100 ppm, which is the natural concentration of phytochemicals found in nectar and is the range suggested to provide therapeutic effect in honey bees and other pollinators [24,26];
- Crude extracts may in fact contain unwanted animal toxins and it is then possible that the compound of interest cannot be confidently removed from the malefactor. For example, many plants, including rhododendrons and others, are known to carry compounds that are toxic to bees (https://bee-health.extension.org/are-there-plants-that-produce-nectar-that-is-poisonous-to-either-honey-bees-or-humans/).

### 2.2. Compound Qualification

It is most practical to perform live bee studies only for compounds that pass some set of criteria and have enough justification for further research, like the aforementioned studies on modified porphyrins. There are two processes to qualify a compound for study in live bees. One is when *ex vivo* studies are simply not possible or practical. Otherwise, one performs in vivo studies when those compounds meet *ex-vivo* potency criteria.

### 2.3. Metrics of Success

A key metric of success of a screened natural product is stable or increased honey bee survival. In parallel, high-throughput qPCR assays are critical to measure transcriptional changes of honey bee genes involved in immunity, lifespan, nutrition or general health and/or decreased pathogen loads. Additionally, non-molecular techniques such as microscopy (e.g., count the number of *Nosema* spores) can be used to measure pathogen loads. For these measures, we know of no community standard on what quantitative value qualifies a compound to receive further consideration as a medicine.

Traditional drug discovery makes use of the therapeutic index, which is the ratio of the dose that produces toxicity to the dose that produces a clinically desired or effective response in a population of individuals [50]. It is calculated as TD_50_/ED_50_, where TD_50_ is the dose of drug that causes a toxic response in 50% of the population and ED_50_ is the dose of drug that is therapeutically effective in 50% of the population. TD_50_ and ED_50_ are determined by supplying a dilution series of doses to honey bees and recording pre-determined measures of toxic response and therapeutic effect at each dose. This data is made into a graph. As far as we know, for honey bees, there is no standard measure of toxic response, although death is a crude measure of toxicity and qPCR is a crude measure of general health (each one is not a ‘be all and end all’, yet each is an acceptable method). Regarding the therapeutic effect, one may measure success, by the gene expression of immune genes or of vitellogenin; measure disease titers, or use other physiological tests (sugar content, respiration, phenoloxidase activity, as some examples).

Here we propose to test one or at most two concentrations initially, which is not enough to determine a dose response by dilution. Only highly desirable candidates should receive the full-fledged dilution series. Others may consider in lieu of the above to measure only endpoint mortality (i.e., deaths not counted daily and only at the end of the experiment) as a crude measure of toxicity. Indeed, this method is the easiest and may be the most practical when one is handling several dozens, if not over one hundred, bee-rearing cups simultaneously. Overall, we know of no honey bee study that has applied a therapeutic index and thus, for our protocol we suggest death and gene expression data as relative measurements of success.

### 2.4. Testing Compounds Without Artificial Pathogen Inoculation (Phase 1a)

#### 2.4.1. Apiary Collection of Worker Honey Bees for Cup Trials

When testing compounds in live honey bees, it is standard to use adult female worker bees. A primary experimental decision to make is whether to use mature (adult) workers or newly emerged workers (NEW). Each approach has its advantages and disadvantages.

Collecting adult honey bees, and more specifically foragers, is our first choice and our recommended research design. First, it is the easier of the two approaches to conform to high-throughput screens. Further, while it does rely on the serendipity of a natural colony infection, very few collections lack key honey bee pathogens (e.g., members of the DWV group). In contrast, we use young bees if our goal is to experimentally manipulate pathogen or symbiont loads, or to otherwise control for confounding factors such as worker age or presence or absence of co-infection. Use of young bees more closely controls for age and enables us to inoculate with specific amounts and types of microbes at specific time points. Emerging bees are typically clean with respect to *Nosema* and *Lotmaria* and possess generally lower titers of DWV, and so we may experimentally manipulate single or multiple infections. Likewise, emerging bees will show few members of the gut microbiome since this community is largely acquired from nestmates [51]. Whether one uses adult or young bees, it is advisable to collect bees from at least two or three colonies. Use of multiple colonies may average out environmental conditions or infection levels, and it may sample a genetic background that differs with respect to resistance or susceptibility to the target pathogen. It is important to record which colonies were used as sources for each replicate.

Collecting adult worker honey bees as in ‘Cup: Mature Bees’ from Palmer-Young et al. [24]:

1. Choose two to three healthy honey bee colonies which, based on prior genetic screens have an appropriate abundance of pathogens for which treatments are to be tested.;
2. Take the outermost honey frames which tend to have older worker honey bees and are less likely to host queen bees;
3. Shake a frame covered with worker bees forcefully into a plastic bucket or alternate container with steep sides >25 cm tall;
4. Immediately shake bees from the bucket into ventilated plastic cups and cap these cups, with about 600 bees/16 oz. (ca. 473 mL) cup. Keep cups of bees in the shade after collection, or ideally in a cooler with an adjacent ice pack on hot days;
5. After transporting the bee cups into the laboratory, anesthetize the cupped bees with CO_2_ for ca. 30 s using a 20 × 20 × 20 cm Styrofoam™ shipping container into which CO_2_ is provided from a tank using surgical tubing. CO_2_ is preferred to placing the bees on ice. In our experience, recovery is >99% versus 80% when bees were chilled;
6. From the cup of anesthetized bees, quickly distribute bees into rearing cups, allocating 30 to 40 bees per rearing cup;
7. A video of this process is provided in the supplement.

Collecting newly emerged worker honey bees as in ‘Cup: Young Bees’ from Palmer-Young et al. [24]:

1. Choose multiple healthy honey bee colonies;
2. Take one or two brood frames from each colony. Select frames that show signs of emerging worker bees to ensure a supply of young bees within one to two days. Place the frames in a screened frame-holding box or a container with a mesh lid;
3. Transport the frames to a laboratory incubator that has been pre-set to 34° C with high relative humidity (50%);
4. Allow worker bees to naturally emerge from the comb within the wire mesh frame cage. One can expect at least 1000 bees from a half-filled brood frame with many, but not all, of the bees emerging within two days. By having multiple combs, one can ensure a sufficient number of bees within one day and the remaining brood can be put back into the colony;
5. Once young bees have emerged, distribute by hand into single-use, semi-sterile plastic rearing cups with 15 to 30 bees per cup.

#### 2.4.2. Experimental Chambers

Variations of plastic ‘bee cups’ [52] are used for all of our trials. While arguments can be made for other chambers, we feel the combination of low-cost and disposability of these cups is critical for contaminant-free assays with hundreds of replicates. We use clear 16 oz. (ca. 473 mL) ‘Solo’ single-use clear drinking plastic cups with a lid and a cross-style straw opening. We trim a plastic ‘Pasteur’ pipette until 0.5 cm of the elongated section is left with the bulb (Figure S1).

We recommend a feeding solution of 50% sucrose dissolved in dH_2_O (weight/volume; herein referred to as 50% sucrose solution), sterilized by autoclaving at 120° C for 15 min. The bees are to be incubated at 32–34 °C with high relative humidity, essentially as with the brood frame.

#### 2.4.3. Treatment Conditions

At the start and on subsequent days, feed bees ad libitum by refilling the pipette bulb with 50% sucrose solution. Untreated control bee cups receive plain 50% sucrose solution throughout the experiment. Treatment cups receive a similar 50% sucrose solution, but with dissolved natural products. The first experiment should start with one concentration of the natural product (100 ppm) or 1% of the crude extract. Incubate the bees for 12 days and record the final number of cumulative deaths at the end of the trial. For the early trials, we generally observe a response, if any, by ten or twelve days. Therefore, for simplicity in Phase 1a, we suggest an incubation duration of about 10 to 14 days. This part of Phase 1a, therefore, tests compounds at one concentration for efficacy using naturally infected worker honey bees. Bees to be artificially inoculated with a pathogen for testing are described later in ‘Testing compounds with artificial pathogen inoculation (Phase 1a)’.

#### 2.4.4. Example Trial

A minimal trial of one natural product at one concentration requires 12 bee cups. Six bee cups are untreated controls and six bee cups are treated with a natural product. Under some circumstances, we also test three cups per condition, especially if we are testing on a whim. A typical natural product concentration is 100 ppm or 1% solution in 50% sucrose solution. The number of bees that a small-scale trial of this magnitude requires is 360 to 480 frame-captured adult worker bees or 180 to 360 newly emerged worker bees, divided among the twelve rearing cups.

#### 2.4.5. Preserving Specimens

Frequently when conducting cup or field trials, we find all laboratory personnel are fully occupied with the trials. This leaves no capacity to immediately perform the RNA extractions, which are labor intensive. Fortunately, it is possible to store frozen bees for weeks or months prior to RNA extraction, without a loss of integrity. We typically remove accumulated dead bees from the cups, directly freeze cups of live bees at −80 °C for at least one hour, and then count and empty cups into marked mesh extraction bags for storage for up to two months. A stress response to the cold by the honey bee should be anticipated if one is not flash freezing the bees with, e.g., liquid nitrogen; however, the use of a control compensates for such responses and, in our laboratory, liquid nitrogen is not readily available.

#### 2.4.6. Output

Upon completion of the cup trial, the next step is to measure pathogen loads and gene expression of honey bee immune genes, as indicators of honey bee health (health for our purposes includes immune-and physiology-related genes). This requires total RNA extraction, first-strand cDNA synthesis, and RNA transcript quantification using 10 to 20 surviving bees taken from one bee-rearing cup. One total RNA isolation from pooled bees from one cup, which reduces ‘cup effects,’ will produce one qPCR (biological) replicate. This is described in ‘Interpretation from Phase 1’.

### 2.5. Testing Compounds with Artificial Pathogen Inoculation (Phase 1a)

#### 2.5.1. *Nosema* as a Targeted Parasite

*Nosema* is an obligate microsporidian fungal parasite that honey bees ingest either accidentally from fecal matter or by feeding. The following protocols are based on standard methods for *Nosema* research. Complete infection dose (ID_100_) for *Nosema ceranae* is 10^4^ spores per bee [53]. Therefore, one can achieve almost complete infection success by inoculating healthy bees with about 10,000 spores per bee. Nevertheless, scientists often feed bees a large excess of spores in order to ensure infection. For a laboratory honey bee trial, it is necessary to have a supply of isolated *Nosema* spores at a known concentration, (freshly) collected from field colonies. Here is one such protocol:

1. To acquire *Nosema* from local apiary, collect 30–50 live foragers from the entrance of colonies that one knows or suspects to carry *Nosema;*
2. *Nosema* infects the honey bee gut; therefore, after collection, the next step to isolate *Nosema* spores is to eviscerate honey bee abdomens. Perform whole intestine extractions as described in [54]. The simplest method is to sedate bees (not by freezing, ideally using CO_2_), and then to pull the entire intestine out by holding the last abdominal segment with a pair of tweezers and slowly pulling until the entire intestine is removed from the abdomen;
3. Combine ten guts in a 1.5 mL sterile centrifuge tube. Add 400 μL of sterile dH_2_0 and grind using a sterile pestle;
4. From this mixture, aliquot 5–10 μL onto a standard glass microscope slide;
5. Place the slide on a microscope and use 400× magnification with phase contrast to look for spores. With phase contrast, if *Nosema* is present, one will observe ovate structures that are distinguishable from any other irregular-shaped debris (Figure S5);
6. Once you identify spores, wash the crude suspension using a modified triangulation protocol 2.2.4.2.1.2. described in [53]. Centrifuge the crude suspension at 300× g for 2 min, remove the supernatant, suspend the pellet in 400 μL of sterile dH_2_0, do a very quick spin of this in a small tabletop centrifuge, and remove the supernatant into a fresh tube. This tube will now contain a more purified spore suspension. Repeat this process at least two more times. In our experience, the quick tabletop spin allows for additional debris to pellet and the spores to remain in the supernatant. Count the spores using a hemocytometer. Keep the spores in water and do not dilute the entire extraction in sugar water;
7. Calculate 10,000 spores per bee and add this number of spores to a volume of 50% sucrose solution when ready to inoculate honey bees. For example, in a cup of 30 bees, 300,000 spores would be suspended in 0.5–1 mL sugar water (cup top feeder):
  - If there are enough spores, then one can proceed to the feeding stage;
  - If one does not have enough spores, one can survey more bees and repeat the protocol;
  - If time allows, feed the suspension created from step 2 to newly emerged bees. Rear the bees using the protocol described above for at least 12 days and then repeat steps 2 through 7 to isolate and count the spores from the bees that were artificially fed *Nosema*. This should generate tens to hundreds of millions of spores depending on how many bees were inoculated, which is definitely enough for a typical trial.

With respect to storage and viability, *Nosema* spores in water suspension remain viable for at least one month when the suspension is refrigerated at 5–7 °C. One should not freeze spores, the suspension or the bees themselves. Field-collected honey bees may carry *Nosema ceranae*, *Nosema apis*, or conceivably, both. It is not always reliable to distinguish between these two species by microscopy alone. Therefore, to determine the relative quantities of *N. ceranae* and *N. apis* in the prepared suspension, one should draw an aliquot from the suspension and perform a simple DNA extraction, and then use diagnostic PCR with species-specific primer pairs as outlined in [53].

#### 2.5.2. Live Bee *Nosema* Screening

With an adequate supply of *Nosema* in hand, it is now time to infect and screen live bees. The *Nosema*-specific protocol is very similar to the standard protocol. Note that there is no type strain for *Nosema* and labs usually isolate *Nosema* locally for their experiments.

1. Prepare the *Nosema ceranae* suspension (above);
2. Collect adult worker bees or newly emerged bees as described in ‘*Apiary Collection of Worker Honey Bees for Cup Trials*’;
3. After collection of the bees, feed the bees with sterile 50% sucrose solution. Incubate at 34 °C with 50% relative humidity (as above) for one day;
4. On the second day, inoculate the adult bees “cage-style” with the suspension from step 1. Using a low volume (likely 500 μL) that contains 10,000 spores for each bee in the cup ensures complete infection and that the bees consume the entire solution within one day [53]:
  (a) If using newly emerged bees, one has the option to do “cage-style” feeding or hand inoculations (see Table S1 in the supplement for cage-style and hand-style feeding success). The food source should be removed in the morning and in the late afternoon, feed 5 μL to each bee using a 10–20 μL pipettor and by holding the bee by its wings and letting the bee drink from the inoculum droplet;
  (b) On the other hand, foragers will consume this amount of food quickly and the feeder might need to be topped off with extra food;
5. After complete consumption of the inoculum, start the compound-sucrose solution feeding at 100 ppm for half of the cups and maintain ad libitum feeding throughout the trial. Replenish as needed;
6. Incubate and dose the honey bees for at least twelve more days to ensure complete infection. It takes at most six days for *Nosema* to complete its life cycle and after twelve days, high mortality is then observed in cup-reared honey bees;
7. Count and record the number of deaths daily and/or at the end of the trial;
8. At the end of the experiment, remove dead bees (which are left in the cups throughout), freeze the remaining live bees at −80 °C, and then transfer the bees into marked extraction bags or tubes to prepare for total RNA isolation;
9. Store bags in a freezer at −80 °C until ready to perform RNA extraction.

Note that *Nosema* infection should not cause a sharp drop in survival until around day twelve post-inoculation. Because of this, sharp mortality early in the experiment may be due to toxicity of the tested compound, an interaction between chemicals and infection, or because the bees have high viral loads or were from a mite-infested brood frame.

### 2.6. Serratia

*Serratia* is an opportunistic bacterial pathogen that appears common in the intestine of honey bees. *Serratia* spp. can negatively affect the longevity of the honey bee [10]. This *Serratia*-specific protocol is like the *Nosema* protocol.

1. Isolate strains of *Serratia* from honey bee intestines as in [55]. Honey bee intestines may be fresh or macerated and stored in 20% glycerol at −20 °C. There is no *Serratia*-type strain for honey bee testing, and the precaution is biosafety level 1;
2. In preparation for the trial, swab the glycerol stock of the isolated and verified *Serratia* strain in LB broth in the evening. Grow overnight, dilute and then incubate to an OD_600_ of about 1.0. Centrifuge the bacteria, wash the pellet with 1X PBS, centrifuge again and suspend in 1× PBS. Combine 20 mL of the bacterial PBS suspension with 50% sucrose solution to make a 25% bacteria-sucrose feed;
3. Collect adult worker bees as described in ‘*Apiary Collection of Worker Honey Bees for Cup Trials.*’ As above, capture honey bees and then incubate the bees for one day with plain sterile 50% sucrose solution;
4. On day two, feed to the bees 1 mL of the *Serratia*-infused syrup and let the bees consume the solution for one day like “cage-style” feeding for *Nosema ceranae*;
5. The next day, start feeding 100 ppm of the test compound in 50% sucrose solution ad libitum to half of the cups;
6. Incubate the honey bees for eight more days. One could expect a sharp increase in mortality after six to seven days;
7. Refer to steps 7–9 in ‘Live Bee *Nosema* Screening’.

### 2.7. Deformed Wing Virus

DWV is one of the most negatively impactful honey bee pathogens known. It has been linked to colony loss through physical deformities, such as wing deformity. There are three widespread variants of Deformed wing virus: DWV-A, DWV-B, and DWV-C. Scientists have sequenced the genomes of all three [56,57]. DWV is endemic in most honey bee populations and it is rare to find an unsullied honey bee or colony [58]. We recommend initial screens for DWV using pan-DWV primers, followed by lineage-specific screens [59]. The prevalence of each strain may fluctuate and some authors report that variant B is replacing previously dominant variant A [57,59].

1. Identify an apiary or colony with relatively high titers of DWV;
  - One may assess colony virus load by collecting 50 adult honey bees and measuring virus load using qPCR as described: “4.3.2. Bulk extraction of RNA from 50–100 whole bees using the acid-phenol method” (omitting the hot phenol step) in [60]. There is no standard for what classifies as a very high or, conversely, very low hive infection to be used for testing; however, by screening various colonies at the apiary, the researchers can determine which hives have relatively higher and relatively lower titers of DWV. This survey need not only be for this trial: the survey provides additional information to the researchers on which hives to use for other experimentation, from which hives to avoid using brood frames, possible recombination trends, and survey data can be passed on to interested parties such as the USDA-ARS BRL;
  - Alternatively, one may feed filtered bee hemolymph containing DWV to bees using the cage-style method (see Table S2 in the supplement);
2. Once a colony with relatively high DWV titers is identified, collect and rear bees as described in ‘Apiary Collection of Worker Honey Bees for Cup Trials’;
3. Feed with sterile 50% sucrose solution and incubate for one day;
4. The next day, start feeding 100 ppm of the test compound in 50% sucrose solution ad libitum to half of the cups;
5. Incubate and treat the honey bees for twelve days;
6. Refer to steps 7–9 in ‘Live Bee *Nosema* Screening’.

### 2.8. Lotmaria Passim

*Lotmaria passim* is a trypanosomatid that honey bee scientists recognize as an emerging pathogen and has recently been linked to poor honey bee health. In our experience, *Lotmaria passim* will not survive in 50% sucrose solution for cage-style feeding. Therefore, one must inoculate newly emerged bees using quick hand feeding with a 20% sucrose solution containing *L. passim*. Our protocol is based on Schwarz and colleagues that was developed in our lab [8,24]. We recommend to purchase the reference-type strain ATCC PRA-422 (ATCC: American Type Culture Collection) as, unlike the aforementioned pathogens, a type strain for testing is available.

1. Process brood frames as described in ‘Apiary Collection of Worker Honey Bees for Cup Trials’ to generate newly emerged worker honey bees;
2. Three days before the experiment, thaw the stock of *L. passim* and inoculate into supplemented DS2 media that additionally contains 5% (*v/v*) fetal bovine serum and antibiotics [8];
3. When ready to harvest on the day of inoculation, pellet the dense culture of *L. passim* in the medium for 10 min at 425× *g*, and suspend it in 1× PBS;
4. Dilute further by 1:100 and count active promastigotes in a Neubauer hemocytometer at 400× magnification;
5. Fix the suspension to a 2000 cells per μL inoculum using 20% sucrose solution (1:1 vol/vol) in 1× PBS;
6. After the newly emerged bees are incubated in the cups for one day, feed the newly emerged bees 5 μL of the L. passim suspension within one day after removing their food source that morning (discard any bee that does not consume the inoculum);
7. Immediately start the treatment of 50% sucrose solution with 100 ppm of the testing compound to half of the cups;
8. Continue rearing the bees until day twelve, whereby after six days, there will be a complete infection detectable by qPCR, at least in the control group;
9. Refer to steps 7–9 in ‘Live Bee *Nosema* Screening’.

### 2.9. Interpretation from Phase 1

#### 2.9.1. Product Toxicity

One measure of compound safety is honey bee mortality during the experiment. When using naturally infected honey bees for testing, if the mortality is greater in the treated versus untreated group, then this suggests that the compound or something in the extract is toxic to the bees. Additionally, if honey bee mortality is much greater in the treated, artificially infected group versus in the untreated, artificially infected group, then this suggests that the natural product is toxic to honey bees or synergistically toxic to the bees only when the pathogen is present. In the secondary screening, we suggest additional controls such as sugar-only controls to narrow down differences between experimental groups. Daily survival can be modeled by a Kaplan–Meier survival curve. As an alternative to Kaplan–Meier, one may use endpoint survival if daily monitoring is too consuming (for example, when doing hundreds of cups in parallel). To calculate the proportion of deaths, simply divide the number of deaths by the total number of bees in the cup from the beginning.

Typically, excess mortality related to product exposure at the above range of doses will disqualify the natural product from further testing. This, however, does not exclude that a lower dosage will be more effective and less lethal. Regardless, unless mortality across all doses is remarkably high, we typically assess pathogen load for all treatments to gain a complete picture of compound effect.

#### 2.9.2. Bee Collections

Our standard is to freeze and preserve 20 bees from each cup. This is possible because usually at least 20 bees survive in each cup. For storage and future RNA extraction, we often split the 20 bees into two extraction bags, 10 bees per bag. This provides us with two RNA extraction determinations per cup, which allows us to measure variation due to differences among extractions. In the instance when less bees are available because of constraints to inoculate many bees (e.g., 15 newly emerged from the beginning), ten bees may be used in one extraction bag to produce one replicate. The RNA isolation protocol also allows for more or fewer bees to be used per extraction bag if the lysis buffer is changed accordingly. As above, we also typically perform at least six cup determinations per treatment. This results in 12 total extraction bags per treatment. Note that we generally do six cups per treatment and two bags per cup with two treatments (6 cups × 2 treatments × 2 duplicates = 24 samples). During statistical analysis of variance, this allows us to partition variation among cups, extractions and treatments. However, as noted above, we sometimes circumstantially reduce the number of cups per treatment to three, and also, sometimes, we will process only one bag per cup; this depends on the amount of resources available at the time.

#### 2.9.3. RNA Extraction

For RNA extraction, we rely on the total RNA extraction detailed in ‘The COLOSS BEEBOOK: molecular methods’ (4.3.2. Bulk extraction of RNA from 50–100 whole bees using the acid-phenol method, omitting the heating of the phenol) [60]. Total RNA for qPCR is stable for years at −80 °C, as is the cDNA. We recently did a quality assurance test on this method and a variation of this method [61]. This is provided in the Supplementary Information (Figure S6).

#### 2.9.4. Measuring Pathogen Load and Honey Bee Health

We measure pathogen load and honey bee health by messenger RNA transcript level using reverse-transcribed RNA followed by quantitative polymerase chain reaction (qPCR). First-strand complementary DNA (cDNA) synthesis is done as described in [60]. Many labs use different cDNA and qPCR reagents protocols and reagents, and tests should be robust. After extraction, qPCR is used to quantify a set of one or more studied pathogens and honey bee reference, immune, and nutrition genes (Table 1).

For consistency, completeness, and comparison among studies, we typically measure this standard set of transcripts plus any other pathogen of interest. Ribosomal protein S5a and Actin-related protein 1 are stable reference genes that allow for adjustment of relative levels, which may differ among specimens or replicates due to differences in starting RNA material quantity. If one reference gene appears variable, likely due to unintended effects from the treatments, then the other reference gene may be used; otherwise, both can be used in combination. Vitellogenin is a nutrition-related gene that is recognized as a general health marker for honey bees and is involved in age and stress. Hymenoptaecin is an antimicrobial peptide gene that is differentially expressed during various infections including *Nosema*, viral, and bacterial.

#### 2.9.5. Analysis of Treatment Effect

The Cq values from the qPCR runs should be exported and opened in a spreadsheet. Calculate the change in cycle threshold (ΔCq = Cq _reference gene_ minus Cq _gene of interest_). The ΔCq represents relative transcript levels, whereby a higher number represents a relatively higher number of transcripts in the specimen. A ΔCq of 1 will indicate twice as many transcripts relative to the reference gene [24]; therefore, if the reference gene is stable across groups, then one can compare the relative number of gene-of-interest transcripts across groups by the ΔCq method. One should do all statistical analyses using the ΔCq. Use the aggregated ΔCqs to test for normality (e.g., Shapiro–Wilk W test). If the data is normally distributed, one can proceed with the most commonly used ANOVA test for an initial examination of whether the ΔCqs of the groups differ. The statistical program will also provide the ΔΔCq between the control and the experimental sample, which will indicate the relative number of transcripts between the control and treatment. Therefore, one compares the means to look for statistically significant differences between the control group and the *in-vivo*-tested compound group. Alternatively, for an initial examination, an equivalent non-parametric test (Wilcoxon test) is more conservative and may be warranted for a low number of biological replicates, even if one is unable to reject normality or if the data is not normally distributed.

After the initial examination, if there is an indication that the ΔCqs of the groups are different, then one can proceed to use a Dunnett’s test (parametric) or a Steel–Dwass control (non-parametric) test to finely examine differences between each treatment group and the sugar-only control group (likely tested in Phase 1b); these tests compensate for multiple comparisons during analysis. Although an internal decision, one can set the alpha level at 0.05 for all cases, and a *p* < 0.05 would be considered a statistically significant difference in the mean ΔCq between two groups. However, we would also recommend following interesting leads if, for example, multiple trials lead to reduced pathogen titers, yet the differences in pathogen load were not statistically significant.

Lastly, the above ΔCq determination method relies on equal amplification of the target across all samples and thus presumes equal primer pair amplification efficiency where the amplicon amount doubles each cycle (denoted in the following equation, where E = 2). One can compensate for differences in primer efficiency using the equation E^ΔCq^, where E = 1 + (%primer efficiency/100) and (ΔCq = Cq _reference gene_ minus Cq _gene of interest_). The resulting value is called normalized relative quantity (N RQ), as described in [8]. In this case, 1 Cq is a twofold change in target template abundance [8].

As an example, using the ΔCq without a primer efficiency calculation (even though the primer pairs have pre-determined efficiencies of 90%–110% as generally accepted), if we have the control with a ΔCq = 1 and the sample with a ΔCq = 2, we already see that the sample has a higher number of transcripts relative to the control. If we use the ΔCq with primer efficiency calculation where the primer pair efficiency is 100%, then we have the control with a N RQ_control_ = 2^1^ = 2 and the sample with a N RQ_sample_ = 2^2^ = 4. We then log_2_ transform the values to the Cq scale to then have N RQ_control_ = 1 and N RQ_sample_ = 2, which brings us to the same results from the ΔCq method. If we compensate for primer efficiency where a primer efficiency was determined to be 90% and using the same ΔCqs for the control and sample example above, then we first get N RQ_control_ = 1.9^1^ = 1.9 and N RQ_sample_ = 1.9^2^ = 3.61. After bringing the values back to the Cq scale by log_2_, we obtain N RQ_control_ = 0.93 and N RQ_sample_ = 1.85. One needs to make their choice of interpreting Cqs before the start of analyses, as with their null hypothesis for the experiment.

Due to the large number of determinations (i.e., all the samples in a treatment) and gene targets to be tested, we recommend running the qPCR reactions as singlets rather than as duplicates. This is, of course, after establishing that qPCR technical replicates do not generally deviate from each other by more than one Cq (Table 2). In our experience, actual biological variation across bees or pooled bees is on the order of several Cq cycles, and this is the variation that is most relevant for testing hypotheses.

## 3. Secondary Screen (Phase 1b)

### 3.1. Compound Selection

Ideally, a promising natural product will emerge from a program of first-pass screens. A promising candidate is one that increases honey bee survival, decreases pathogen load, increases immune gene expression, or a combination of all three. Alternatively, results must be looked at very carefully; indeed, a downregulation of immune gene expression may indicate relief of the required host response whereas upregulation may indicate stimulation. When a promising candidate emerges, it is subject to a second round of laboratory testing using the previously described protocols, but with additional parameters. The purpose of this is to repeat the first screen to assess reproducibility of results, and to gain a more complete picture of dose effects.

### 3.2. Expanded Protocol

The protocol for the second screen expands on the protocol of the initial screen. It repeats the first protocol while adding additional conditions.

1. Repeat the original trial with a new set of rearing cups and source colonies in order to increase the number of biological replicates and statistical power. Use the same dose as the initial trial but add a dose treatment that is ten times lower to get a range of efficacy. A dose-dependent response curve can eventually be assessed;
  (a) For certain natural products, the dose window might be shifted to address concerns about solubility, toxicity or weak impact at a low dose, as determined from in vitro testing;
2. In parallel, run the sucrose-only and compound-only untreated control in order to have additional controls. For example, in the inoculation studies, the first screen had two conditions where all the bees were inoculated with the pathogen yet only one condition was with the test compound. Now, we add two conditions where bees are fed either an unsoiled food source, as well as the compound without the pathogen;
3. As noted above, endpoint survival for the first trial may be sufficient, especially when a large number of bee cups are running in parallel. However, in the second round, it is necessary to monitor survival by counting deaths daily or every other day for three to four weeks (time already incubated included) in order to produce a proper survival curve and to monitor the longevity of the worker bee (e.g., a decline in the *Nosema*-infected population will likely only be seen 12 to 14 days after the start of infection);
4. Feeding the compound from the onset of infection for pathogen-inoculated honey bees follows a similar protocol set up in our laboratory, as described in Palmer-Young et al. [24]. However, variations in the experimental design can include waiting three to six days post-infection before administering the compound of interest. This would allow the pathogen in question to establish itself in the host by giving it time to start its reproductive life cycle.

At the specified time point per assay (the end of experiment for the original trial), take three bees from each cup, totaling 15 biological replicates at low-dose, high-dose, and one control (45 samples in total). Remaining bees in the cups that were not used for total RNA isolation should be labeled and stored in case additional biological replicates are warranted. Proceed with individual bee extractions. Use the ‘4.3.1. TRIzol^®^ extraction’ method set out in [60]. A quality control of this method was also done, and the RNA integrity is provided in the supplement. After extraction, perform first-strand cDNA synthesis and qPCR like above. Run the qPCR in singlets. If worker bees are alive at the end of the extended survival trial, these bees should also be frozen and later analyzed by qPCR. Analyses are as described. Additionally, they may include a dose–response relationship that has a higher resolution of survivorship. One may use Kaplan–Meier methods to construct survival curves and then use log-rank method to test for statistically significant differences between survival curves.

Additional points to consider for secondary screens:

1. All candidate substances that might end up as a bee treatment should be verified for stability in 50% sucrose solution by subjecting it to liquid or gas chromatography coupled with mass spectrometry (LC/GS-MS). This will partially confirm that the natural product in question is still present and whether there was extensive degradation. Additionally, crude materials bought from different companies and under different storage conditions, in which one expects there to be the compound of interest, may in fact have degraded or be nonexistent which would severely reduce or eliminate the material’s efficacy; this can also be confirmed by spectroscopy and mass analyses. If there is an extract is of interest, then one should consider purchasing it as fresh as possible and to monitor its composition overtime;
2. Since this protocol ensures the availability of stable cDNA from each specimen, it may also be informative and economical to measure additional RNA viruses that are detrimental to bee health (such as Lake Sinai virus 2).

In summary, one should assess bees for mortality and levels of gene expression (Table 2) to determine the effect of the natural product on honey bee health and its efficacy to combat honey bee pathogens. At this stage, we would generally disqualify a natural product that even hints at a deleterious effect on bee survival. After consideration of the totality of the results of the secondary screen, one should consider testing the natural product at the colony-level (“field trial”) with similar goals (Phase 2 and Phase 3).

## 4. Small, Local Field Trials (Phase 2)

### 4.1. Mark-Recapture Trials

The next step in vetting a natural product that showed increased survival, decreased pathogen load, and/or increased levels of honey bee nutrition and immune genes (Phases 1a and 1b) is to test the compound in a colony setting (Figure 1: Phase 2). For a preliminary field test, we favor testing the compound by controlled exposure in the lab, followed by residency in a field colony. The goal is to expose treated bees to a natural hive environment where they will acquire a typical microbiota and the most prevalent parasites and pathogens [8]. The following is a summary of a mark-recapture study repurposed for natural product field tests:

1. For source colonies, select three that have a queen (queenright), are of near-equal strength and do not show overt signs of disease:
  (a) This is a judgement call on behalf of the beekeeper. At the simplest level, a queenright colony, with an equal number of Langstroth frame boxes and with healthy brood patterns can be considered similar enough for colony tests. Alternatively, weighing a colony is possible, but this is more intensive;
2. Collect several hundred very young worker bees from each colony in a similar manner and from similar frames;
  - Very young bees on brood frames can be collected gently and quickly by hand or by a modified “insect” vacuum (Figures S3 and S4). In general, collected bees will only differ a couple days in age from each other. The easiest method for identification is whether the bees are attending to the brood and if they are a bit gray in color;
3. In the lab, distinctly mark 50 worker bees per condition using non-toxic paint pens (e.g., ‘Pro-Painter’ pens, generally sold for queen marking);
4. Place marked bees into single-use plastic cups with each cup having a distinct color scheme (using two color spots it is simple to produce up to 16 distinct marking patterns) (Figure S2);
5. Feed bees for 48 h with a 50% sucrose solution treatment containing a natural product or controls using the determined dosage from the second trial in Phase 1b:
  - Compounds can be tested in bees that have pathogens that they already acquired from the colony; or
  - Bees can also be fed *Nosema* spores (10,000 cumulatively per bee) or a suspension of viruses (e.g., 10^9^ copies per bee) at this stage, as desired;
  - The 50% sucrose solution with and without the compound should be immediately fed to the collected and painted bees (optionally, artificially inoculated bee);
6. Mix bees of all colors into a single cup for at least two hours. This improves equal acceptance, we feel, while not offering bees too long to share microbes or carried treatments;
7. Select source colonies and introduce marked bees to this hive via the top frames of the top box. We do not find high mortality in these introductions, perhaps because bees are introduced away from the guarded nest entrance. It is not uncommon to see >95% survivorship at one week;
8. After ten days, collect all marked bees from the colony using a modified portable vacuum (e.g., the BioQuip bug vacuum). Each hive box, if two, must be separated and each frame pulled and inspected on both sides by two people, one of whom is handling the vacuum. Frames are set outside and then returned to the clean box one at a time, again checking carefully for all marked bees;
9. Freeze collected bees at −80 °C. Once frozen, bees can be sorted by color for counting, and placed into color-coded groups for RNA extraction and analyses for disease levels and honey bee transcripts as above.

### 4.2. Small-Scale Colony Trials

1. Select a minimum of 16 colonies (one treatment condition and one control set), which have been equalized, paired by size (worker number, brood area, and stored nutrition, [65]) and randomly placed into either set;
2. Use standard top feeders to deliver products and nutrients. These trials work best when colonies are actively receiving sugar supplements;
3. For each colony, expect to use six liters of 50% sucrose solution in total, three liters per week for two weeks. This provides each bee with about 150 μL of 50% sucrose solution on average, assuming a colony population of 40,000 bees. The bees will store much of the treated 50% sucrose solution, but it remains accessible and the colony should consume it over the next two months;
4. Collect and freeze 300 worker bees per colony after two weeks, one month, two months and finally, on the third month after the start of the trial. This is in addition to collected bees from day 0;
5. At each collection point, additionally screen colonies for colony metrics and any overt signs of disease;
6. Extract RNA from 50 collected bees using a bulk extraction method: e.g., “4.3.2. Bulk extraction of RNA from 50–100 whole bees using the acid-phenol method (omitting the hot phenol step)” [60];
7. Perform qPCR to measure pathogen load and bee health using the set of standard pathogen and host gene assays as described above. Add assays to measure additional pathogen or host genes as desired.

A natural product may fail field trials for safety (toxicity) or lack of efficacy. A compound that is effective in the laboratory may not be in the field for several reasons. One is that controlled cage studies in the lab offer a higher resolution to monitor individual changes in the host. At the colony-level, the compound is diluted amongst the whole superorganism, as well as stored. Another reason is a weak effect that gets swamped out in the colony by natural environmental variation. If a promising natural product fails its first field trial screen, this failure may be due to a changeable condition, such as delivery season, scale of the dose or an unknown factor that led to the colony to die.

## 5. Large-Scale Field Trials (Phase 3)

The final experimental step to demonstrate safety and efficacy is to expand small-scale field trials to numerous colonies, beekeepers and geographic regions (Figure 1: Phase 3). To register a product in the U.S., the regulatory bodies of the EPA and FDA might mandate up to 1000 trial colonies. Achieving this number of colonies is likely to require the cooperation of at least several commercial beekeepers who each maintain thousands or tens of thousands of colonies. Operations of this size usually exist primarily to reap pollination fees for annual crop production and hence, are migratory.

Regardless of colony use, these field trials should be conducted after honey extraction. This is to minimize the chance of contaminating the honey with natural products. Other than that, this larger-scale field trial is similar to the smaller-scale trial. The colonies of the commercial beekeeper will migrate with the beekeeper as they follow their fall pollination season in advance of winter, typically ending in the Southern U.S.A in the secondary screen, field technicians will collect 300 worker bees from each selected and marked colony, after two weeks, at one month and at two months. Generally, the same protocol is followed as previously described, but without using painted bees. These bees can be sent to the Bee Research Lab, or other organizing and capable entity, to assess pathogen load and bee health using the genetic assays, as described above. Beyond natural product testing, such survey data is very precious. Typically, one would also assess colony health once more at three months after the start of the experiment.

## 6. Epilogue

### 6.1. Cooperation

An important aspect of registration is that success will likely depend on the cooperation of at least several entities (Figure 1: Realization). These entities may be a federal laboratory, a university laboratory, backyard beekeeper or major commercial beekeeper. It will be necessary for these entities, and perhaps the community at large, to share data, to manage intellectual property rights, and to cooperate with regulatory agencies for proper assessment of health and safety. This process may only succeed when all partners prioritize, as their primary objective, the development of economical, safe and effective tools for bee health.

Researchers may desire collaboration or assistance from the USDA-ARS Beltsville Bee Research Laboratory (BRL). The Laboratory welcomes inquiries and has often, but not always, found a way to assist the community in unique projects. As a federal lab, we have the resources and capabilities to advance the nation’s interest for crop protection and stability. However, the Lab is unable to endorse a specific product.

### 6.2. Registration

In the U.S., registration is granted by the federal Environmental Protection Agency (EPA) or by the Food and Drug Administration (FDA). The exact agency may depend on the chemical properties of the natural product or its risk of entering the food supply. It is key to engage regulatory agencies early in the process of a specific natural product to assess potential registration challenges.

One may want to consult regulatory groups after a successful small-scale field trial or even after a positive secondary screen. In our experience, the U.S. EPA is motivated to help beekeepers remain viable in the face of diverse threats. This urgency should favor successful registration if the natural product has scientific verification for safety and efficacy.

### 6.3. Promise of the Natural Product Approach

Evidence shows that reducing honey bee virus load and supplementing honey bee immune responses may be possible using natural product extracts from living organisms. Examples include mushrooms; foraged plant material (e.g., nectar, pollen and plant resins); and mixed honey bee-made compounds (e.g., propolis). It is an open question as to whether any of these natural products will demonstrably and consistently improve honey bee populations for agricultural use. The most likely way to find out is through a community effort that takes advantage of harmonized protocols, measures and methods of analysis.

Investigation of the possible positive impact of natural products on honey bee health and controlling disease will improve the community’s understanding of the safety and efficacy of specific natural products and will advance understanding of honey bee biology. Our goal in this paper is to initiate discussion of protocols and workflows that may evolve into a repeatable mechanism for the development and commercialization of safe-for-use honey bee natural product medicines, both therapeutic and perhaps prophylactic.

It is likely that the need to find novel honey bee health products will be ongoing. Researchers will discover and propose new products and honey bee threats will change and evolve. We strongly believe that the investigation of natural products will lead to honey bee medicines that are safe, effective and have a large positive influence on honey bee health, as aspirin, quinine, penicillin, digoxin and taxol have for humans.

Returning honey bee overwinter mortality to sustainable, historical levels will contribute to the long-term profitability of the beekeeping industry. It will help control hive management costs, reduce hive loss and increase hive productivity. In turn, this will lead to higher crop quality and yield, and thereby advance the security of the nation’s food supply.

## Figures and Tables

**Table 1:** qPCR primers for a standard set of measured transcripts.

Disease/Host	Target	Forward Primer	Reverse Primer	Reference
*Lotmaria passim-*specific	*Lotmaria passim*	AGTATGAGCAGTAGGTTTTATTATA	GCCAAACACCAATAACTGGTACT	[62]
Deformed wing virus-	DWV-A,-B	ACGCAACCCCAGGAAT	GTAGCTAATTTTACCCAATCTTTAAA	[63]
Nosemosis	*Nosema ceranae*	TATTGTAGAGAGGTGGGAGATT	GTCGCTATGATCGCTTGCC	[53]
Bacterial infection	Bacteria (all), including *Serratia*	AGAGTTTGATCCTGGCTCAG	CTGCTGCCTCCCGTAGGAGT	[8]
Reference gene (host)	Ribosomal protein S5a (*Rps5a*)	AATTATTTGGTCGCTGGAATTG	TAACGTCCAGCAGAATGTGGTA	[8]
Reference gene (host)	Actin related protein 1 (*Arp1*)	CCAAAGACCCAAGCTCCCTA	TGGCTTATTGGTTTATGTTTTTCGT	[8]
Immunity gene (host)	Hymenoptaecin (*Hym*)	CTCTTCTGTGCCGTTGCATA	GCGTCTCCTGTCATTCCATT	[64]
Age/nutrition/immunity (host)	Vitellogenin (*Vg*)	TCGACAACTGCGATCAAAGGA	TGGTCACCGACGATTGGATG	[8]

**Table 2:** A simulated chart for checking qPCR data. For simplicity, a sample number code can be used on tubes and extraction bags with a spreadsheet containing the precise details. Technical duplicates of qPCR reactions for the specimen are run and the Cq determined by the qPCR software. If the technical duplicates are close to one another, similar to those in Table 2, then one can proceed to qPCR singlet reactions. The reference gene *RPS5a* was run and also the antimicrobial peptide (AMP) gene Hymenoptaecin (“*Hym*”). The ΔCq = Cq *_RPS5a_*minus Cq _gene of interest “*Hym*”_) was calculated. We observe that the honey bees that were inoculated with *Nosema* followed by natural product dosing (+*Nosema*+NP) had a higher relative gene expression of hymenoptaecin than the control treatment of a *Nosema* infection without the natural product dosing (+*Nosema*-NP). qPCR data should be coupled with survival data. CoV: Coefficient of Variation; 1st: first of two technical qPCR reaction replicates; 2nd: second of two technical qPCR reaction replicates; Replicate difference: the Cq difference between each technical qPCR reaction; Average: the average of the two technical qPCR reaction runs (not done when run in singlets); ΔCq: the difference between the reference gene (*Rps5a*) and the target gene (e.g., *Hym*), whereby a higher number represents higher relative gene expression.

**Sample ID#**	**Treatment**	**Replicate**	***Rps5a*** **1st**	***Rps5a*** **2nd**	**Average**	**Replicate Difference**	**CoV**	
1	+*Nosema*+NP	1	20.19	19.75	19.97	0.44	1.557971876	
2	+*Nosema*-NP	1	21.1	20.03	20.565	1.07	3.67908707	
**Sample ID#**	**Treatment**		***Hym*** **1st**	***Hym*** **2nd**	**Average**	**Replicate Difference**	**CoV**	**ΔCq**
1	+*Nosema*+NP	1	25.2	26.1	25.65	−0.9	2.481076425	−5.68
2	+*Nosema*-NP	1	28.2	28.9	28.55	−0.7	1.733711898	−7.985

## Supplementary Materials

The following are available online at https://www.mdpi.com/2075-4450/10/10/356/s1: Figures S1: Worker honey bees housed in bee cups for assays, Figures S2: Painted bees returned to a common cup prior to release, Figures S3: Frames removed as they are checked for painted bees to collect. After the inside of the box is scanned, frames will be returned one by one, slowly checking each side, Figures S4: Hand insect vacuum (BioQuip) fitted with a short length of surgical tubing for collection of individual bees, Figures S5: Phase contrast microscopy pictures of extracts from the intestines of worker honey bees that were artificially infected with *N. ceranae* spores, Figures S6: Bioanalyzer runs of a total RNA extraction from three different total RNA extraction methods. Tables S1: Nosema spore feeding results, Tables S2: DWV feeding results.
