# Supplementary material for: Natural Product Medicines for Honey Bees: Perspective and Protocols
Source: Insects. 2019 Oct 18;10(10):356. doi: 10.3390/insects10100356 (PMC6835950; doi:10.3390/insects10100356)
Supplement: Supplementary file 1 [file insects-10-00356-s001.pdf]

Supplement: Natural product medicines for honey bees: Perspective and protocols

James P. Tauber[1*], William R. Collins[2], Ryan S. Schwarz[3], Yanping Chen[1], Kyle Grubbs[1], Qiang Huang[4], Dawn Lopez[1], Raymond Peterson[1,5], and Jay D. Evans[1*]

[1] USDA-ARS Bee Research Laboratory, BLDG. 306, RM. 315, BARC-EAST, Beltsville, MD 20705, USA

[2] Department of Chemistry, Fort Lewis College, 1000 Rim Drive, Durango, CO 81301, USA

[3] Department of Biological Sciences, Fort Lewis College, 1000 Rim Drive, Durango, CO 81301, USA

[4] Honeybee Research Institute, Jiangxi Agricultural University, Zhimin Avenue 1101, 330045, Nanchang, China

[5] Granite Point Ventures LLC, 10 Lakeview Circle, Greenbelt, MD 20770, USA

[*] correspondence: Jay.Evans@usda.gov (J.D.E.); james.tauber@usda.gov (J.P.T.)

Bee cup rearing design

This is supplemental information for '*Apiary Collection of Worker Honey Bees for Cup Trials: Experimental chambers*' that is found in the main text.

For all incubator trials, bees were maintained at 32^0C in standard rearing incubators with a standing tray of water to increase humidity (Figure S1). Field-collected bees for exposure in the laboratory were collected as shown in https://youtu.be/c7utyUzWgQ4.

For the mark-recapture assays, bees were painted on the thorax with distinctive colors, returned after treatment to a common container for two hours (Figure S2) and then released to host colonies. At the close of the experiment marked bees were collected using a hand vacuum (Figures S3 and S4).

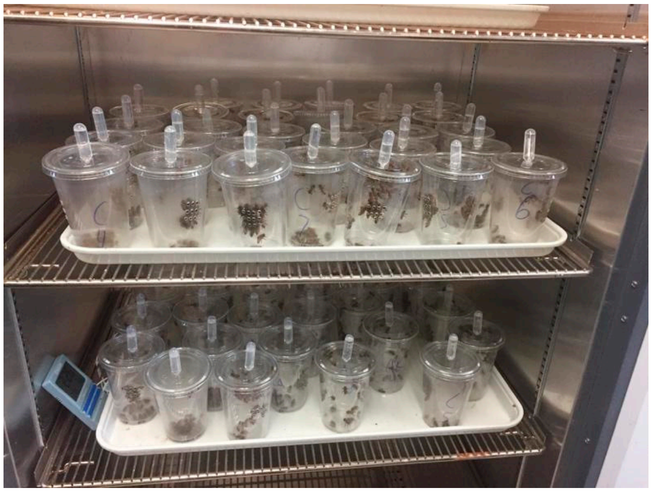

Figure S1. Worker honey bees housed in bee cups for assays.

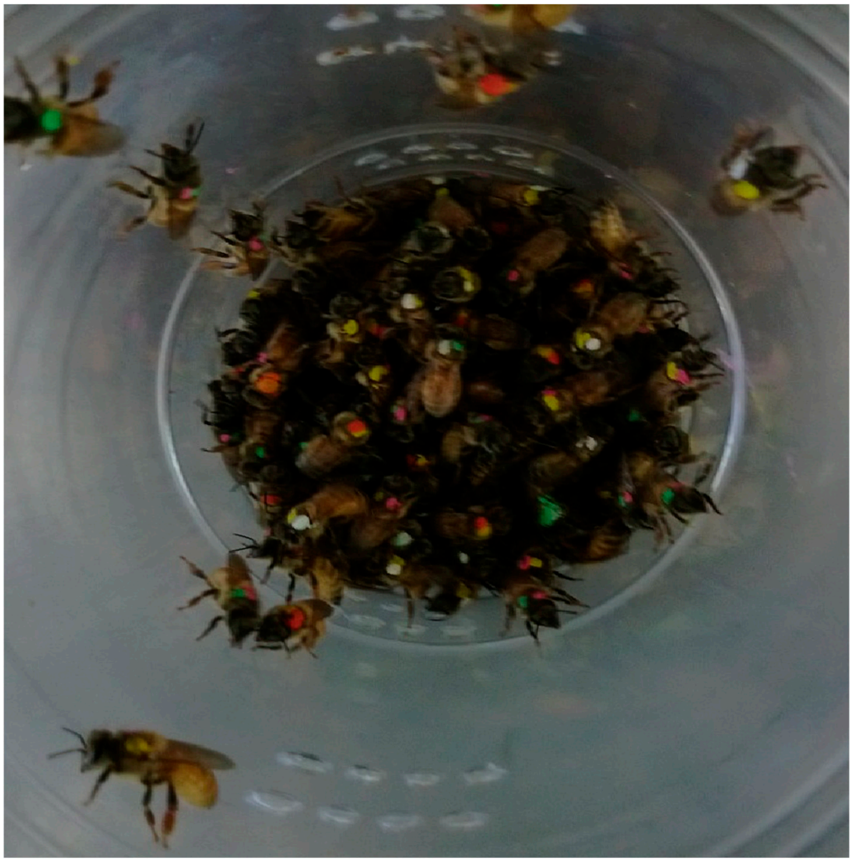

Figure S2. Painted bees returned to a common cup prior to release.

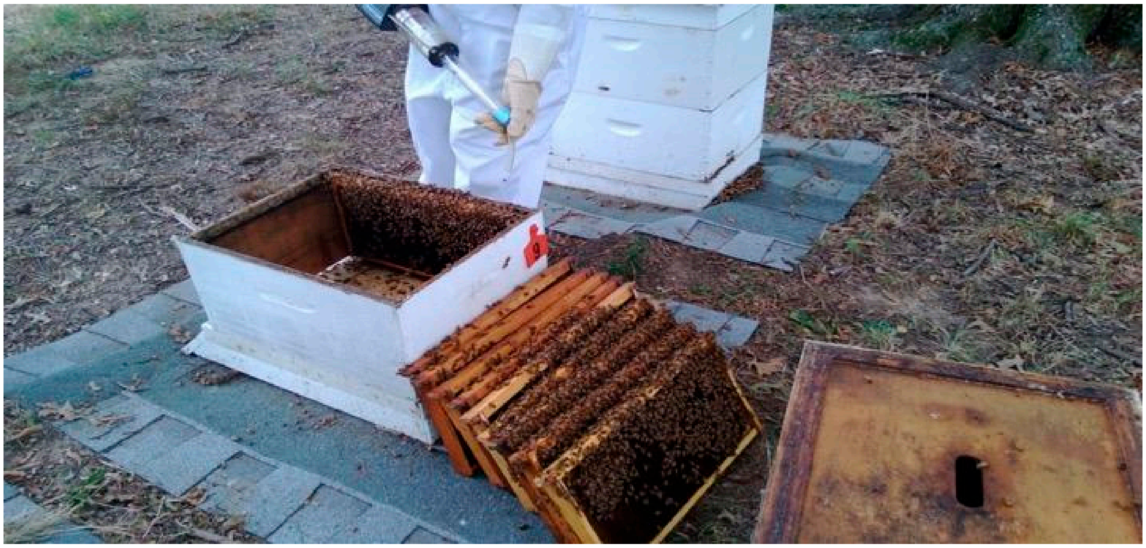

Figure S3. Frames removed as they are checked for painted bees to collect. After the inside of the box is scanned, frames will be returned one by one, slowly checking each side.

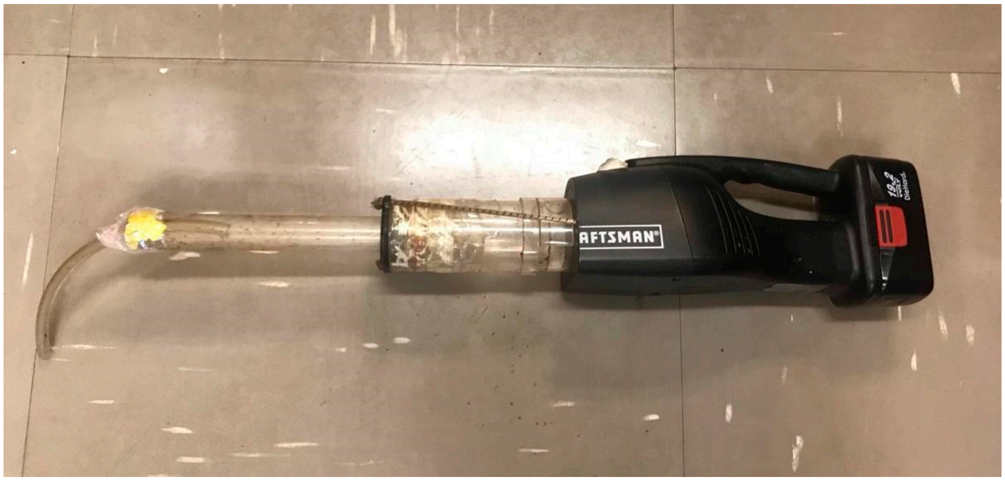

Figure S4. Hand insect vacuum (BioQuip) fitted with a short length of surgical tubing for collection of individual bees.

Nosema spore suspension preparation

This is the supplemental information to '*Testing compounds with artificial pathogen inoculation (Phase 1a):*

Nosema as a targeted parasite' that is found in the main text.

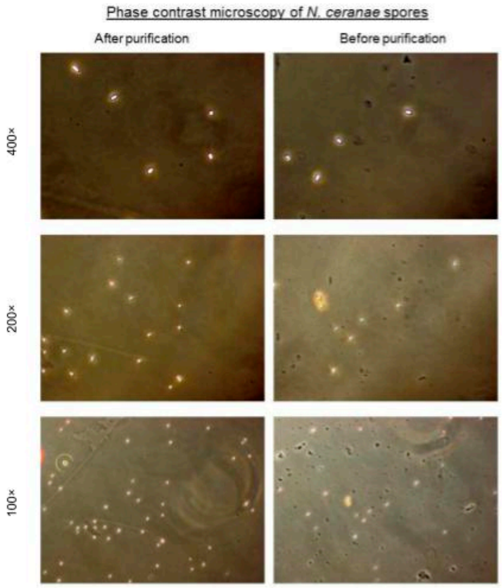

Figure S5. Phase contrast microscopy pictures of extracts from the intestines of worker honey bees that were artificially infected with *N. ceranae* spores. 5 µl of the extract was spotted onto a standard glass microscope slide which was overlaid with a cover slip. Spores were washed and purified as described in the main text and we present microscopy pictures at 100×, 200× and 400× of both before and after purification. Pictures were taken by first using the 40× objective to focus on an area, and then using a lower linear magnification while at the same location.

Nosema spore feeding results

This is the supplemental information to '*Testing compounds with artificial pathogen inoculation (Phase 1a):* Live Bee *Nosema* Screening' that is found in the main text. Here we present a small subset of unpublished results. We fed young worker bees 5 µl of the *Nosema ceranae* suspension by hand, totaling 10,000 spores per bee. We also cage-style fed young bees, which also totaled 10,000 spores per bee. Total RNA was extracted from bees using TRIzol® (Thermo). We did qPCR as described in [1]. The purpose of this was to show that after ten days post *N. ceranae* feeding there was a relatively higher spore load of *N. ceranae* in the intestine and that the fungus was active as determined by cDNA template derived from *N. ceranae* mRNA transcripts. Therefore, we present the success of the *Nosema* feeding styles, rather than constancy of molecular biology techniques. We present aggregated Cq values in the following table.

**Hand or cage style inoculation**	**Gene**	**Treatment**	**Average Cq**	**Standard deviation Cq**	**Median Cq**	**Number samples**
Hand	*Arp1*	+*Nosema*	23.384	1.343974915	23.48	15
		-*Nosema*	23.20272727	0.881976087 1	23.07	11
		Both (data combined)	23.30730769	1.153691667	23.255	26
	Ncer	+*Nosema*	32.18533333	10.70827708	28.34	15
		-*Nosema*	50	0	50	11
		Both (data combined)	39.72230769	12.03224179	45.595	26

**Hand or cage style inoculation**	**Gene**	**Treatment**	**Average Cq**	**Standard deviation Cq**	**Median Cq**	**Number samples**
Cage	*Rps5*	+*Nosema*	23.01	0.688734749 8	23.02	10
		-*Nosema*	23.825	0.362008287 2	24	10
		Both (data combined)	23.4175	0.679387301 4	23.495	20

	Ncer	*+Nosema*	19.978	1.768689408	19.54	10
		-Nosema	50	0	50	10
		Both (data combined)	34.989	15.4489938	36.705	20

Table S1. qPCR Cq values are presented here. For the hand-fed experiment, a Cq of one sample was produced by qPCR using cDNA synthesized from total RNA extract of one whole intestine from either *N. ceranae*-fed bees or the control group (bees were harvested 10 days post infection). For the cage-fed experiment, a Cq of one sample was produced by qPCR using cDNA synthesized from total RNA extract of one whole abdomen from either *N. ceranae*-fed bees or the control group (bees were harvested 14 days post infection). We present aggregated Cqs from replicates for the reference gene (*Arp1* or *RPS5*) and also from a primer pair specifically targeting *N. ceranae*. If there was no amplification, a default Cq value of 50 was given which is the last cycle of the qPCR run.

DWV feeding results

This is the supplemental information to '*Testing compounds with artificial pathogen inoculation (Phase 1a):* Deformed Wing Virus' that is found in the main text. Here we present a small subset of unpublished results. We fed young worker bees a hemolymph-derived DWV suspension (in sucrose and PBS) cage-style. Total RNA was extracted from one abdomen using TRIzol® (Thermo). We did qPCR as described in [2]. The purpose of this was to show that after seven days post DWV feeding there was a relatively higher DWV load in the bee. Therefore, we present the success of the DWV cage-style feeding, rather than constancy of molecular biology techniques. The young bees that naturally emerged from the comb cells were already infected with DWV-A; therefore, this method to inoculate DWV can elevate the DWV-A titers in bees if needed. We present aggregated Cq values in the following table.

**Hand or cage style inoculation**	**Gene**	**Treatment**	**Average Cq**	**Standard deviation Cq**	**Median Cq**	**Number samples**
Cage	Actin	+DWV	19.94875	0.779042402 7	19.85	12
		-DWV	20.59375	1.129704896	20.405	12
		Both (data combined)	20.27125	1.013782941	20.085	24
	DWV	+DWV	22.45875	5.596733762	25.54	12
		-DWV	31.16958333	7.179386239	33.085	12

		Both (data combined)	26.81416667	7.741137371	27.57	24

Table S2. qPCR Cq values are presented here. For the DWV cage-fed experiment, a Cq of one sample was produced by qPCR using cDNA synthesized from total RNA extract of one whole abdomen from either DWV-fed bees or the control group (bees were harvested seven days post infection). We present aggregated Cqs from replicates for the reference gene actin and also from a primer pair specifically targeting DWV-A.

Total RNA Extraction using the BEEBOOK method

This is the supplemental information to '*Interpretation from Phase 1:* RNA Extraction' that is found in the main text. For quality control of the BEEBOOK bulk, whole bee, bag RNA extraction method, we did multiple extractions and then measured the quality of the total RNA isolate both spectroscopically and by qPCR. This was done in our recently published work and the results presented here are data therefrom [1]. From multiple extractions using the BEEBOOK bulk total RNA extraction methodology, we obtained the following information by spectroscopy and qPCR (mean and the standard deviation from eight replicates): ng/µl: 1209.79(235.73); $A_{260/280}$: 2.07(0.08); $A_{260/230}$: 1.38(0.09); *RpS5* Cq: 25.78(4.14); *Vg* Cq: 27.38(4.79); and DWV Cq: 25.08(5.04). The RNA content was considered intact as determined from Bioanalyzer runs. Here we provide an electropherogram from one RNA isolate (Sample 7 in Figure S6) as well as the total RNA quality from RNA extracted from one individual whole worker bee using the BEEBOOK lysis buffer method (Sample 10 in Figure S6). We also present the total RNA quality from an individual whole worker bee total RNA extraction using the TRIzol® (Thermo) method (Sample 4 in Figure S6). Therefore, the BEEBOOK method is versatile and includes total RNA extractions of individual bees in addition to pooling whole bees.

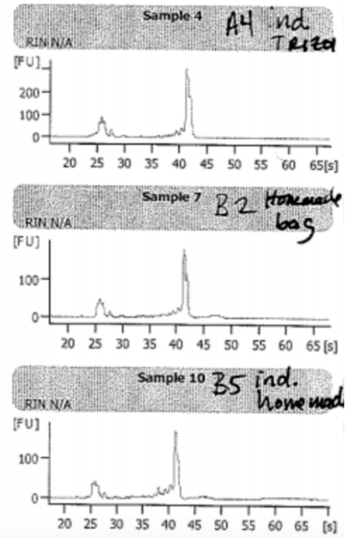

Figure S6. Bioanalyzer runs of a total RNA extraction from three different total RNA extraction methods.

References

[1] Tauber, J.; Nguyen, V.; Lopez, D.; Evans, J.D. Effects of a resident yeast from the honeybee gut on immunity, microbiota, and nosema disease. *Insects* **2019**, *10*, 10.3390/insects10090296.

[2] Palmer-Young, E.C.; Tozkar, C.O.; Schwarz, R.S.; Chen, Y.; Irwin, R.E.; Adler, L.S.; Evans, J.D. Nectar and pollen phytochemicals stimulate honey bee (Hymenoptera: Apidae) immunity to viral infection. *Journal of Economic Entomology* **2017**, *110*, 1959-1972, 10.1093/jee/tox193.
